# Catalyzed Reaction of Cellulose and Lignin with Methyltrimethoxysilane—FT-IR, ^13^C NMR and ^29^Si NMR Studies

**DOI:** 10.3390/ma12122006

**Published:** 2019-06-23

**Authors:** Joanna Siuda, Waldemar Perdoch, Bartłomiej Mazela, Magdalena Zborowska

**Affiliations:** Institute of Wood Chemical Technology, Faculty of Wood Technology, Poznań University of Life Sciences, Wojska Polskiego 28, 60-637 Poznań, Poland; waldemar.perdoch@up.poznan.pl (W.P.); bartlomiej.mazela@up.poznan.pl (B.M.); magdalena.zborowska@up.poznan.pl (M.Z.)

**Keywords:** cellulose, lignin, wood hydrofobization, catalyst reaction, silanization, aluminium acetylacetonate

## Abstract

It can be found that reaction mechanisms and interactions between wood and organosilicone compounds have not been sufficiently explored. The aim of the study was to determine bonds formed between either cellulose or lignin and methyltrimethoxysilane (MTMOS) during a catalytic silanization reaction. Silanization was performed in the presence of two catalysts of a diverse mechanism of functionalization: aluminum acetylacetonate (Al(acac)_3_) and acetic acid (AcOH). For this purpose, FT-IR, ^13^C and ^29^Si NMR techniques were used. Cellulose silanization efficiency without a catalyst was unlikely. Lignin undergoes a silanization reaction with alkoxysilanes much easier than cellulose. The results showed new bonds between biopolymers and the silanising agent. The new bonds were confirmed by signals at the FT-IR spectra, e.g., 770 cm^−1^ and 1270 cm^−1^ (Si–CH_3_), and at the NMR signal coming from the T^1^, T^2^ and T^3^ structures. Efficiency of reaction was confirmed by atomic absorption spectroscopy (AAS) analysis.

## 1. Introduction

In recent years, in the literature there has been a growing interest in the silanization reaction application as far as an increase in wood hydrophobicity is concerned. It can be found in these same sources that reaction mechanisms between wood and organosilicone compounds have not been sufficiently explored [1,2,3,4,5,6]. It has not been verified that the bonds (including expected covalent bonds) between wood and organosilicone compounds are formed regardless of selected reaction conditions. It is known that silanization reaction content is influenced by silanized material water content, catalyst presence, pH, applied organosilicone compounds and solvents. Increasing alkoxysilanes hydrolysis reaction content with the use of catalysts and appropriate pH may result in developing stable bonds between either cellulose or lignin and alkoxysilanes. Dry lignocellulose materials are not of high reactivity for alkoxy groups present in alkoxysilanes. However, only a little amount of water in wood may lead to alkoxy group hydrolysis, thus increasing the affinity between organosilicone compounds and cellulose [7,8]. It is crucial to hydrolyze alkoxysilanes to silanols in order to create bonds between cellulose and alkoxysilanes. The developed silanols are more reactive to hydroxy groups than alkoxysilanes. The reaction may result in developing bonds between cellulose fibers and organosilicone compounds. Forming the bonds may be a desired effect, as it will influence the improvement of wood properties, including hydrophobicity, resistance to fungi and dimensional stability [9,10].

Lignin, next to cellulose, is the second most significant wood component of a polymer character. However, its chemical structure is more complex than cellulose. The structure is constituted by C–O–C and C–C bonds, and its fundamental construction unit is a phenyl-propane group [11,12]. Lignin is more likely to be reactive to alkoxysilanes than cellulose due to the hydroxy groups’ presence in phenol groups. Lignin can form two types of bonding with alkoxysilanes: Si–O–C and hydrogen bonds [13]. The likelihood stems from the fact that phenol groups are more electrophilic and thus more reactive [7].

Organosilicone compounds, such as trialkoxysilanes (of a general chemical formula R’Si(OR)_3_, R-alkyl group), including alkoxy groups, may be hydrolyzed [14,15,16]. In order to catalyze and improve reaction content, the following catalysts can be applied:

- complex compounds (aluminum acetylacetonate, chromium (III) acetylacetonate);
- complex compounds of metal alkoxides (complexes of aluminum sec-butanolan);
- acids (acetic acid, hydrochloric acid) [17,18,19,20].

Zhang and Sakka [18] employed metal β-diketonates to catalyse the hydrolysis reaction of selected alkoxysilanes. These compounds catalyze the hydrolysis reaction of methyltrimethoxysilane (MTMOS) in solution and they also affect their polymerization. Regarding this, using aluminum acetylacetonate to catalyze cellulose and lignin silanization in the presence of alkoxysilane—which is methyltrimethoxysilane (MTMOS)—was attempted in the article.

**Figure 1:**
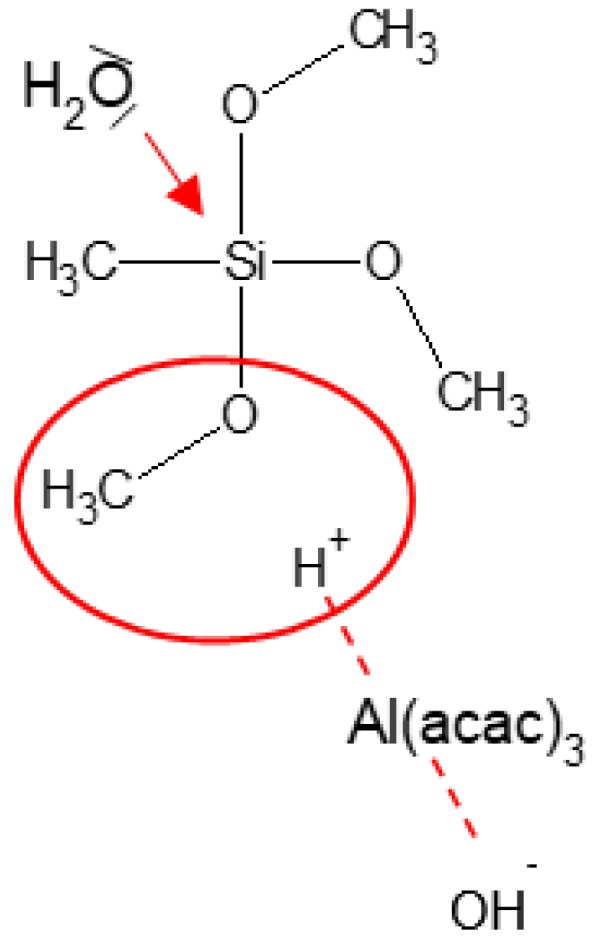
Transition state developed in solution according to alkoxysilane hydrolysis mechanism with applying aluminum acetylacetonate (Al(acac)_3_) [18].

According to the literature, alkoxysilanes hydrolysis in the presence of aluminum acetylacetonate should theoretically occur according to the alkali mechanism as the complex is alkali. In fact, the presence of aluminum acetylacetonate (Al(acac)_3_) results in substituting a ligand with a water molecule, and a protonated transition state is present in the solution (Figure 1). An alkoxy group is protonated by receiving a proton from an intermediate of H_2_O and Al(acac)_3_, and hydrolysis occurs by an attack of another molecular H_2_O on silicon, forming a leaving group of ROH [18]. It is worth emphasizing that even in neutral pH, the catalysis of Al(acac)_3_ leads to protonation of alkoxy groups, in the consequence of which a hydrolysis reaction occurs.

**Figure 2:**
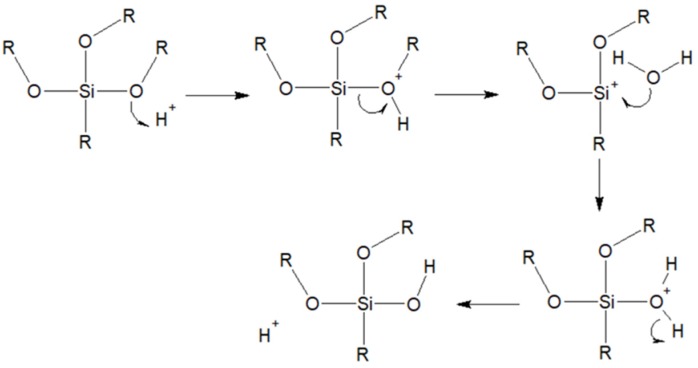
Alkoxysilane hydrolysis mechanism in an acid environment; R = alkyl [22].

Wood silanization has been performed mainly in an acid environment, i.e., in accordance with the acid hydrolysis mechanism, up until now [21]. The acid functionalization mechanism is based on the protonation of an alkoxy group. Proton transforms the RO^−^ group into a good leaving group, and its place is taken by a water molecule with a positive charge on an oxygen atom, after which the reaction environment is restored (Figure 2).

The aim of this study was to determine bonds formed between either cellulose or lignin and MTMOS during catalytic silanization reaction with Al(acac)_3_. Silanization was performed in the presence of two catalysts of a diverse functionalization mechanism—aluminum acetylacetonate (Al(acac)_3_) and acetic acid (AcOH). The bonds formed during silanization were determined with the techniques of infrared spectroscopy (FT-IR), as well as carbon and silicon nuclear magnetic resonance spectroscopy (^13^C and ^29^Si NMR). FT-IR was applied in order to determine the silanization reaction content through measuring band intensity, whereas the NMR analysis was applied in order to confirm bond formation between either cellulose or lignin and MTMOS. Atomic absorption spectroscopy (AAS) analyses were used to analyze silicone content.

## 2. Materials and Methods

### 2.1. Materials

The following materials were used in the research: cellulose (C) as powder with a grain diameter of 50 µm (Sigma Aldrich, CAS no 9004-34-6, S. Louis, MO, USA) and Kraft lignin (L) as powder (Sigma Aldrich, CAS no 8068-05-1, S. Louis, MO, USA). Kraft lignin had been selected because of its pH (pH = 6.5), being near neutral, in order not to affect a reaction trend, i.e., hydrolysis according to an acid mechanism.

Methyltrimethoxysilane (MTMOS) (Sigma Aldrich, CAS no 1185-55-3, S. Louis, MO, USA) was used as a silanization agent for cellulose and lignin. Aluminum acetylacetonate (Al(acac)_3_) (Sigma-Aldrich, CAS 13963-57-0, S. Louis, MO, USA) and acetic acid (AcOH) (POCH, CAS no 64-19-7, Gliwice, Poland) were selected as catalysts for the silanization reaction. Ethanol (POCH, CAS no 64-17-5, Gliwice, Poland) was used as a solvent.

### 2.2. Silanization

The reaction mixture consisted of MTMOS (9.5 g), solvent (7.9 g ethanol), a catalyst and either cellulose (1 g) or lignin (1 g). The following number of catalysts was used in the research: 0.45 g (2 × 10^−2^ mol per 1 mol of alkoxysilane) of Al(acac)_3_ and 0.21 g (0.05 mol per 1 mol of alkoxysilane) of AcOH. Corresponding proportions had been employed by Brinker [17] and Zhang and Sakka [18], who had investigated hydrolysis of MTMOS itself. The detailed content of the investigated reaction mixture is shown in Table 1. The reagents were mixed (24 h, 500 rpm, 22 °C) and centrifuged (20 min, 2500 rpm, 22 °C). Then, the centrifuged mixture was separated through decanting of the solution over cellulose or lignin. The obtained reaction products were conditioned in a desiccator with colloidal silicon dioxide (up to constant mass).

In order to evaluate the durability of the silanization, leaching was carried out. The samples were subjected to washing with distilled water (three times, 30 min each). Next, cellulose or lignin was filtered on a Buchner funnel and dried in a desiccator with colloidal silicon dioxide (up to constant mass). The leached samples were marked with “W”.

### 2.3. FT-IR Analysis

FT-IR spectra were obtained by means of a FT-IR spectrometer produced by Bruker Optics GmbH (Ettlingen, Germany). Powder samples of treated cellulose or treated lignin (2 mg) were dispersed in a matrix of KBr (200 mg), followed by compression to form pellets. The sample collection was obtained using 32 scans, in the range of 4000 to 400 cm^−1^, at a resolution of 4 cm^−1^.

Bands at 897 cm^−1^ [23] and 1510 cm^−1^ [24,25] were selected as stable bands of cellulose and lignin, respectively. Bands at 777 cm^−1^, 1270 cm^−1^, 1032 cm^−1^, 1127 cm^−1^ and 3450 cm^−1^ were selected as characteristic bands for silanized cellulose and lignin. On the basis of an intensity ratio of basic bands and characteristic bands observed on FT-IR spectra, the silanization reaction content was determined.

The IR spectra were used for the calculation of the lateral order index (LOI, H1430/H898), hydrogen bond intensity (HBI, A3340/A1336) and total crystalline index (TCI, H1372/H2900) according to literature data [26].

### 2.4. NMR Analysis

Solid sample measurements were performed with a BRUKER Avance III 400 spectrometer (Ettlingen, Germany), at a resonance frequency of 100.63 MHz for ^13^C and 79.495 MHz for ^29^Si, equipped with a magic angle spinning (MAS) probe combined with 4 mm ZrO_2_ rotors. The bands were recorded at the sample rotation speed at a magic angle spinning (MAS) amounting to 8000.

A ^13^C CP-MAS (cross polarization magic angle spinning, (Ettlingen, Germany) spectrum was performed according to the following analysis conditions: 90° proton pulse with the length of 6.0 μs, 90° pulse for ^13^C 11 µs, a contact time of 2 ms and a relaxation delay of 4 s. Data acquisition was conducted in the spectrum range from 40.25 kHz (400 ppm) recording a FID (free induction decay) signal of 11.8 Hz/pt point density (in matrix 4K data points), as well as proton decoupling with a SPINAL16 sequence applied during acquisition. A glycine sample was employed to optimize the Hartmann–Hahn condition and chemical shift pattern (δC=O = 176.50 ppm).

A ^29^Si CP-MAS (cross polarization magic angle spinning, (Ettlingen, Germany) spectrum was performed using the following analysis parameters: 90° proton pulse with the length of 6.0 μs, 90° pulse for ^29^Si 7 µs, contact time of 5 ms and a relaxation delay of 4 s. Data acquisition was conducted in the spectrum range from 48 kHz (604 ppm) recording a FID (free induction decay) signal of 11.8 Hz/pt point density (in matrix 4K data points), as well as proton decoupling with a SPINAL16 sequence applied during acquisition. The Q8M8 sample was employed to optimize the Hartmann–Hahn condition and chemical shift pattern (δ = 0.00 ppm).

### 2.5. AAS Analysis

Samples of approximately 0.1 g were weighted and placed in a clean Teflon vessel with 65% HNO_3_ and 48% HF (Merck, Darmstadt, Germany). Digestion was carried out using a Microwave Reaction System: Multiwave PRO equipped with the acid digestion rotor 8NXF100. The concentration of Si was determined using the Shimadzu AA7000 F-AAS analytical technique (Shimadzu, Kyoto, Japan).

## 3. Results and Discussion

### 3.1. FT-IR Analysis of Silanized Cellulosesilanized

**Figure 3:**
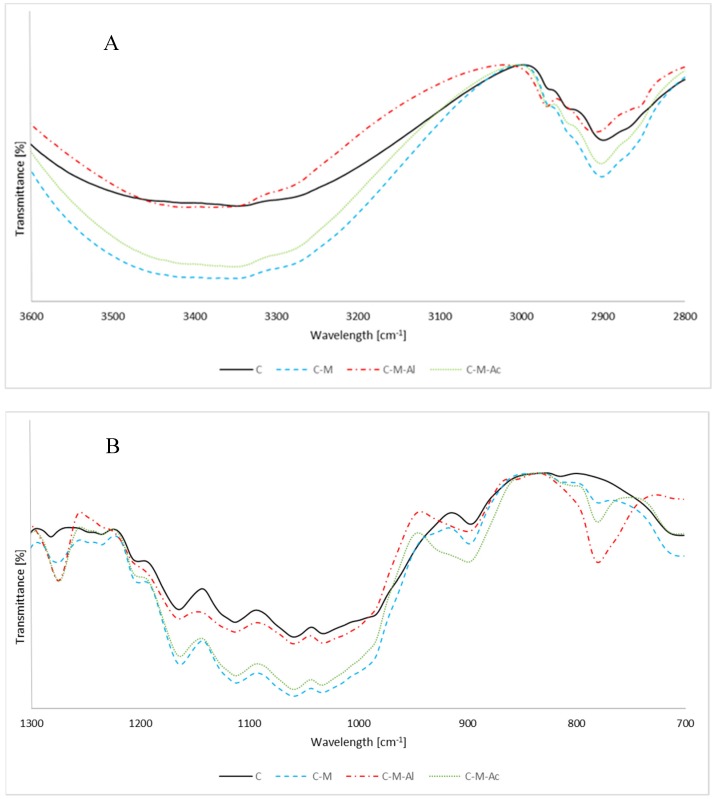
FT-IR spectra of silanizated cellulose at the 3600 to 2800 cm^−1^ range (**A**), and at the 1300 to 700 cm^−1^ range (**B**).

The FT-IR spectra of cellulose silanizated according to the variants C, C–M, C–M–Al and C–M–Ac are presented at Figure 3.

A band at 1127 cm^−1^ corresponding to stretching vibrations Si–O–Si (overlapping with C–O–C vibrations [27]) was observed on each spectrum of cellulose silanized with MTMOS [28]. As a result of cellulose silanization performed in the presence of catalysts (C–M–Al and C–M–Ac variants), new bands at approximately 770 cm^−1^ and 1270 cm^−1^, which corresponded to Si–CH_3_ bond vibrations, appeared on the spectra [29]. Occurrence of the aforementioned signals may prove the formation of bonds between cellulose and MTMOS. The presence of bands resulting from Si–O–Si bonds suggests that silanization reaction, as a result of hydrolysis followed by condensation between hydrolyzed MTMOS molecules, occurred in the reaction mixture. At 1032 cm^−1^, a band resulting from Si–O vibrations was observed (overlapping with C–O vibrations [27]). It is worth emphasizing that band intensity observed on silanized cellulose spectra varied in each case.

The calculated cellulose silanization reaction content is presented in Table 2. The observed band intensity for cellulose silanized in the presence of Al(acac)_3_ (C–M–Al variant) was the highest, and the ratio of 770 cm^−1^ and 1270 cm^−1^ band height to a basic band (897 cm^−1^) was 1.67 and 2.14, respectively. The highest intensity is reflected in the fact that the highest reaction content was achieved in the cellulose silanization variant. There was also an observed slight effect of acetic acid on cellulose silanization (C–M–Ac variant). The ratio of band height of 770/897 cm^−1^ was 0.49, whereas for cellulose silanized without catalysts (C–M variant) it was 0.38. The band ratio of 1270/897 cm^−1^ for the C–M and C–M–Ac samples was at a similar level, and amounted to 1.34 and 1.29, respectively. The low intensity of bands characteristic for Si–CH_3_ bonds may mean low efficiency of silanization for the C–M and C–M–Ac samples.

There was a significant increase in intensity of bands within a hydroxy group band (3360 cm^−1^) for cellulose silanization without catalysts (C–M variant). The increased band intensity may stem from overlapping cellulose hydroxy group bands and groups formed after alkoxysilane hydrolysis. The intensity ratio of –OH band to C–H band was 6.53. A similar phenomenon occurred for silanization with acetic acid (C–M–Ac variant), and the ratio was 4.03. The lower intensity of the 3360 cm^−1^ band, which was observed on a silanized cellulose spectrum, could mean the occurrence of Si–OCH_3_ hydrolysis and the polymerization of developed silanols. The observation above implies the formation of new bonds between cellulose hydroxy groups and the developed silanols and their polymers.

For Si–O and Si–O–C bands (signals at 1032 cm^−1^ and 1127 cm^−1^) the ratio to a basic band was the highest for the C–M sample. The high intensity of those bands resulted from the presence of bonds between cellulose and hydrolyzed MTMOS and condensed MTMOS, which could precipitate on the cellulose surface.

In order to evaluate properties of the crystalline structure of cellulose, the LOI, TCI and HBI were calculated. The results of this test are presented in Table 3. The decrease in LOI and TCI indicates that the reaction degrades the cellulose crystallites and its ordered structure, and the proportion of amorphous to the crystalline region increases. The increase in HBI may indicate the presence of hydroxyl groups come from MTMOS hydrolysis and condensation reaction. Based on these results, it can be said that this is a bulking and surface reaction.

### 3.2. FT-IR Analysis of Silanized Lignin

**Figure 4:**
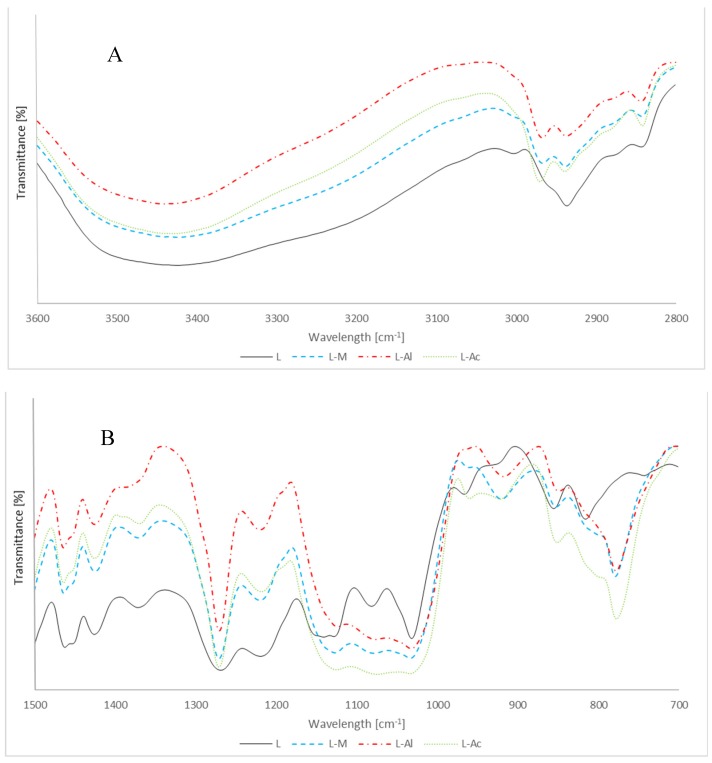
FT-IR spectra of silanizated lignin at the 3600 to 2800 cm^−1^ range (**A**), and at the 1500 to 700 cm^−1^ range (**B**).

The FT-IR spectra of silanizated lignin are presented in Figure 4. The signals at 777 cm^−1^ and 1266 cm^−1^ corresponding to Si–CH_3_ vibrations, as well as 920 cm^−1^ corresponding to Si–OH vibrations, were observed on the reaction product spectra without a catalyst.

This may be attributed to higher lignin reactivity resulting from its chemical structure, namely, from the presence of more reactive hydroxyl groups than in cellulose. Lignin, in its structure, contains phenol groups whose proton is of high acidity. These are phenol groups which can be the place for forming bonds with alkoxysilanes.

In order to compare lignin silanization content to MTMOS in the presence of catalysts, the intensity ratio of band characteristic intensity was calculated (Table 4). For lignin, a band selected as stable was a band at 1510 cm^−1^, corresponding to C–H vibrations of an aromatic nucleus. Lignin silanized in the presence of acetic acid was the most reactive, which is reflected in the highest coefficient of band intensity corresponding to Si–CH_3_ vibrations, and amounted to 0.89 and 1.73 for bands of 770 cm^−1^ and 1266 cm^−1^, respectively. It is worth adding that in the case of L–M where a catalyst was not applied, coefficients were at an equally high level, which meant the reaction was occurring without a catalyst.

### 3.3. Cellulose ^13^C, ^29^Si NMR Study

**Figure 5:**
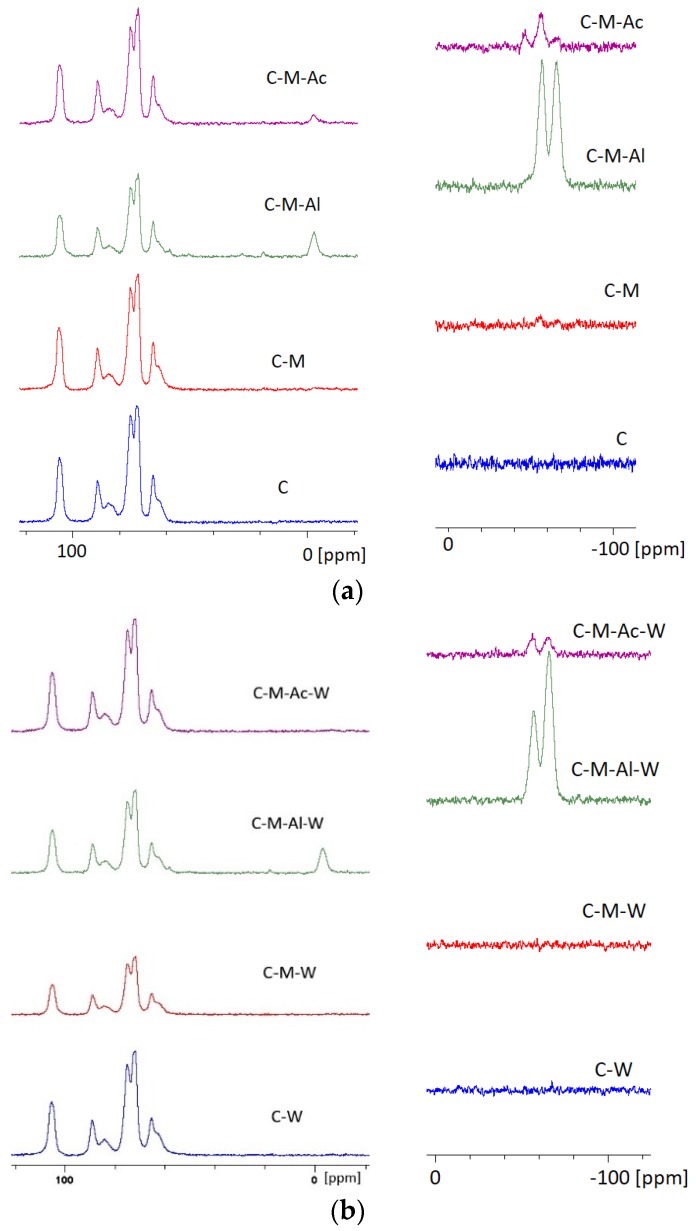
Spectra: (**a**) ^13^C and ^29^Si NMR of cellulose; (**b**) ^13^C and ^29^Si NMR of cellulose after leaching.

Figure 5 presents the set of spectra of ^13^C NMR and ^29^Si NMR of cellulose and silanized cellulose before and after leaching. According to the literature, the following signals were attributed to a particular carbon atom in a cellulose molecule: C1—105.49 ppm; C2, C3, C5—range 70.00–78.00 ppm, C4—84.61 ppm; C6—65.50 ppm [30,31]. The same peaks were observed after leaching for samples C–W (Figure 5b).

No alternations in comparison to a pure cellulose spectrum were observed on the spectrum of ^13^C NMR of cellulose silanized with MTMOS (Figure 5a). Moreover, peaks characteristic for bonds with a silicon molecule were not noticed on the ^29^Si NMR spectrum of silanized cellulose. Only minor signals were observed at −55.88 ppm (near-noise signal). The signals may correspond to linear groups called T^2^ structures in the scientific literature [32]. In the silanization variant in which the reaction was conducted in the presence of an Al(acac)_3_ catalyst (Figure 5a), significant differences were observed. On the spectra of ^13^C NMR, three new signals occurred at 58.52, 18.60 and −3.10 ppm. The signals correspond to carbon atoms of a Si–O–CH_2_ methylene group (stemming from cellulose), a Si–O–CH_3_ methoxy group (present in MTMOS) and a –CH_3_ methyl group, respectively [33]. The presence of those signals could mean the presence of silicone compounds in the cellulose structure [32]. On the other hand, on the spectrum of ^29^Si NMR (Figure 5a), intense peaks were observed at 57.04 and −66.10 ppm. The peaks represent siloxane linear groups (−57.04 ppm), as well as more complex cross-linked structures with three siloxane bonds (−66.10 ppm). The specialist literature calls the above signals T^2^ and T^3^ silane structures, respectively [4,32,34,35,36]. The presence of the aforementioned signals and, on the other hand, the lack of signals representing monomers in which a silicon atom is not bound with another silicon atom (so-called T^0^ structure) and signals stemming from a Si–Si dimer (so-called T^1^ structure) may imply that aluminum acetylacetonate catalyzes effectively a cellulose silanization reaction with MTMOS. After leaching, the disappearance of signals at 18.60 ppm and −3.10 ppm in the ^13^C NMR spectrum was observed (sample C–M–Ac–W). The disappearance of said signals indicates the leaching residues of unbound MTMOS. However, the same peaks (such as before leaching) were observed for samples C–M–Ac–W and C–M–Al–W (Figure 5b) on spectra ^29^Si NMR after leaching. When a reaction was catalyzed with acetic acid, on a silicon spectrum a signal of −45.43 ppm corresponding to a dimer structure of MTMOS, it was observed that the signals representing linear and spatial silane groups (T^2^, T^3^ structures) were considerably less intense than signals observed on the spectrum of silanization reaction with an Al(acac)_3_ catalyst. On a ^13^C spectrum, a peak at −2.95 was observed, representing a methyl group presented in MTMOS. Nevertheless, signals appearing after the application of aluminum acetylacetonate were not observed (58.52, 18.60 and −3.10 ppm). The lack of the signals above may mean a lowered hydrolysis reaction content and condensation.

### 3.4. Lignin ^13^C and ^29^Si NMR Study

**Figure 6:**
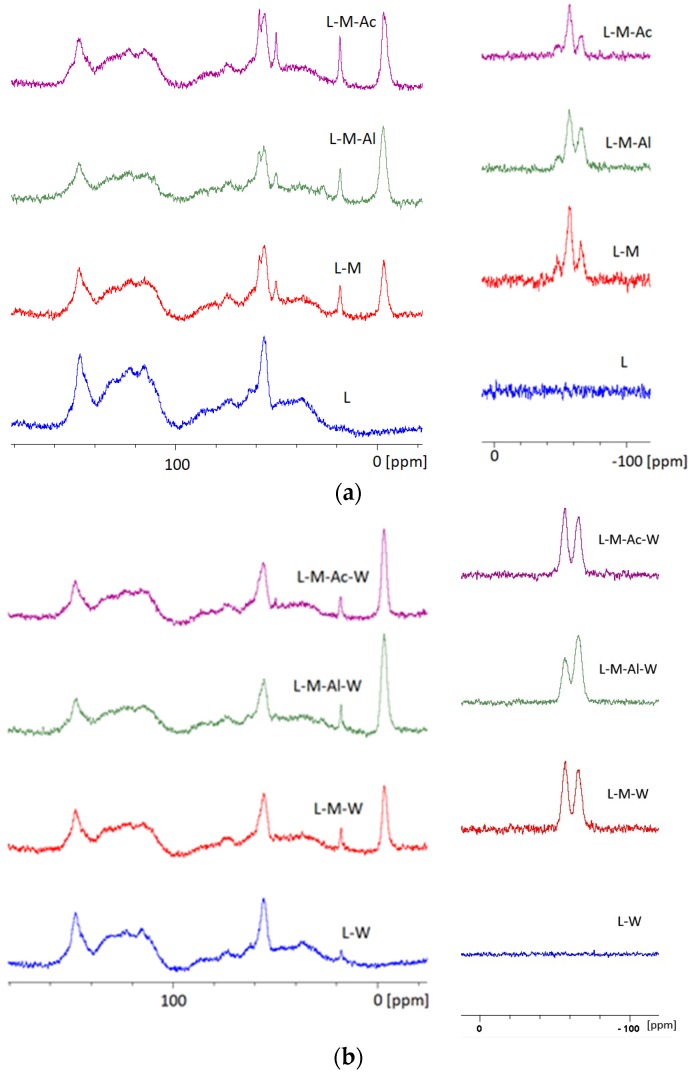
Spectra: (**a**) ^13^C and ^29^Si NMR of lignin; (**b**) ^13^C and ^29^Si NMR of lignin after leaching.

Figure 6 presents the set of spectra of ^13^C NMR and ^29^Si NMR of lignin before and after leaching. Peaks at 147.16 ppm, 121.77 ppm and 115.48 ppm can be attributed to carbon atoms C4, C6 and C5, respectively, in guaiacyl structure and were identified on the ^13^C NMR spectrum of unmodified lignin. Peaks at 72.10 and 68.99 ppm represented carbon atoms in the α, β and γ location. Carbon in a methoxy group occurred at 55.98 ppm [37,38,39].

On the ^13^C NMR spectrum of silanized lignin without a catalyst (Figure 4b), new signals at 58.32 ppm, 18.02 ppm and −3.84 ppm were observed. The signals correspond to carbon atoms in Si–O–CH_2_, Si–O–CH_3_ and CH_3_ groups [33,40]. For the same sample, signals −47.68 ppm, −57.14 ppm and −65.62 ppm representing dimers, linear links and more complex structures (T^1^, T^2^ and T^3^ structures, respectively) were observed on the ^29^Si NMR spectrum. On the ^29^Si NMR spectrum, being the image of the sample of lignin silanized in the presence of aluminum acetylacetonate (Figure 6a), linear structures and more complex T^2^ at −56.54 ppm and T^3^ at −65.60 ppm, respectively, were observed. After leaching, the same peaks were observed for samples L–M–W, L–M–Ac–W and L–M–Al–W (Figure 6b). Signal loss from monomers (T^1^ structure) implies that MTMOS silanization reaction content in the presence of Al(acac)_3_ is higher than for silanization without a catalyst.

For lignin modified in the presence of acetic acid (Figure 6a), the NMR research results were comparable with lignin research results without a catalyst. On the ^13^C spectrum, signals at 58.33 ppm, 18.42 ppm and −3.21 ppm representing carbon atoms in Si–O–CH_2_, Si–O–CH_3_ and CH_3_ groups were observed. On the ^29^Si NMR spectrum, signals from dimers, linear structures and more complex structures occurred (T^1^ at −47.90 ppm, T^2^ at −57.40 ppm and T^3^ at −65.70 ppm, respectively). 

### 3.5. AAS Study

Table 5 shows results of silicone content measured with AAS. The highest content of silicone in the case of cellulose (61.49 mg/g) before leaching was observed for the sample C–M–Al. This result confirmed the fact that the high efficiency of silanization was obtained in the presence of Al(acac)_3_. A significantly lower Si content was observed for the C–M and C–M–Ac samples, where the values were 3.60 mg/g and 34.94 mg/g, respectively. The C–M–Al–W sample was characterized by an increase in the content of Si. The silicon content in this sample was 70.02 mg/g. The increase in the silicon content could be related to the fact that during the leaching there was further reaction of MTMOS with cellulose.

The highest silicon content for modified lignin (84.07 mg/g) was observed for the L–M–Ac sample. Lower content of silicon was observed in the samples L–M–Al and L–M, where the content was 71.72 mg/g and 61.22 mg/g, respectively. This result confirmed that it was not required to use the catalyst for the effective lignin silanization. The increase in the silicon content for the L–M–Al–W sample was observed. It is most likely that the increased silicon content resulted from the fact that the water used for leaching and the remains of Al(acac)_3_ promoted the silanization reaction with unreacted MTMOS. In the other samples (L–M–W and L–M–Ac–W) after leaching, the silicon content decreased by 5.6% and 9.0%, respectively. These results confirmed that lignin was highly reactive to MTMOS and the formed bonds were durable. 

### 3.6. Mechanism of Bonding

**Figure 7:**
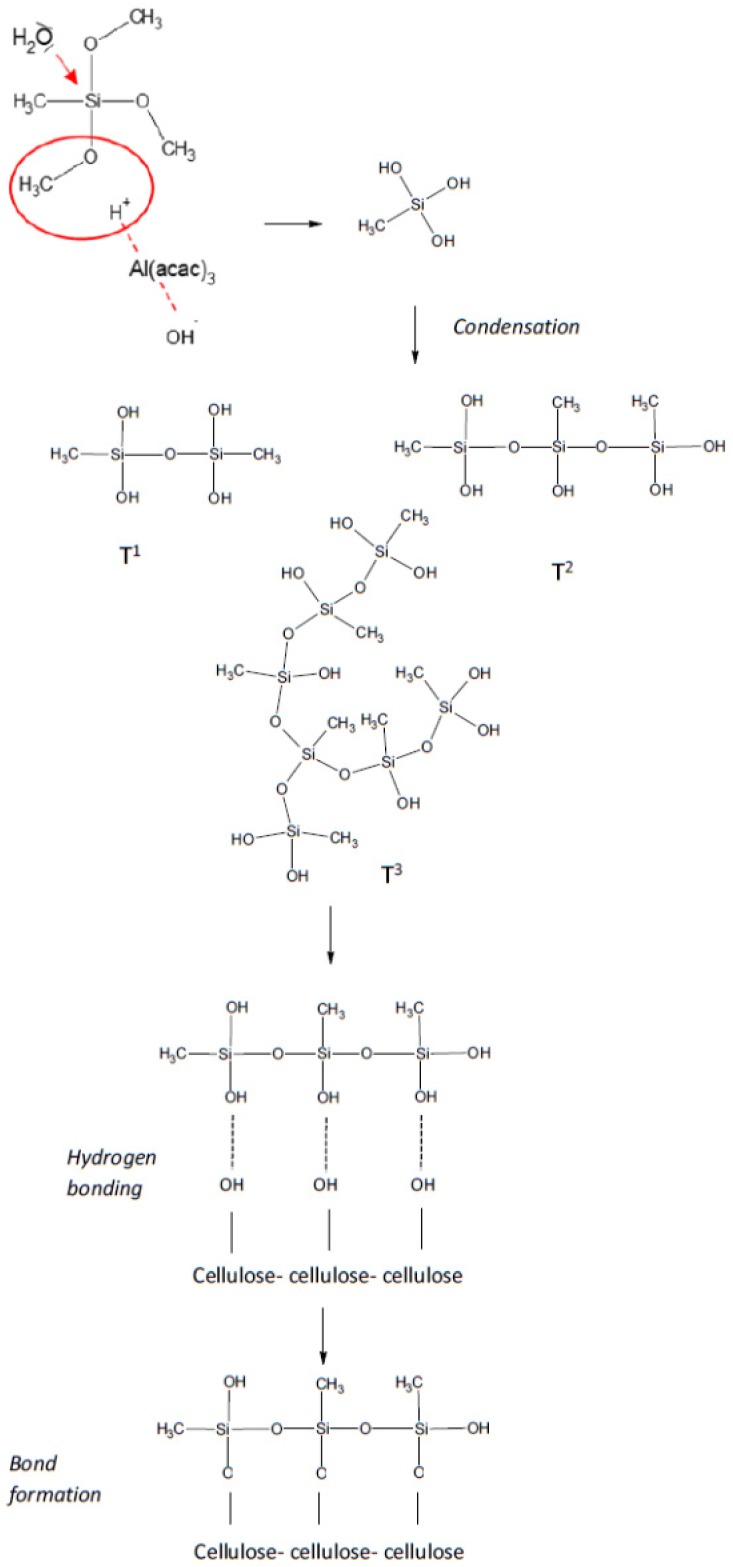
Presumed mechanism of MTMOS bonding to cellulose.

Based on the results of FT-IR and NMR, it was proven that a catalyst was needed for cellulose silanization. The highest efficiency of silanization was obtained for the reaction in the presence of Al(acac)_3_. A feasible silanization mechanism of cellulose in the presence of Al(acac)_3_ has been proposed at Figure 7. The first step of the reaction was the hydrolysis of –OCH_3_ groups. Hydrolysis run in a presence of Al(acac)_3_. A ligand substituting with a water molecule and a protonated transition state was present in the solution. A methoxy group was protonated by a proton stemming from a water molecule transition state and Al(acac)_3_, and hydrolysis occurred via the attack of another water molecule on an alkoxy group. As a result of the reaction, a methoxy group formed a group leaving –OCH_3_. After that, the condensation reaction was running. Products of this reaction was confirmed by NMR analysis where, T^1^, T^2^ or T^3^ structures were observed. These structures formed hydrogen bonds with OH groups on the surface and inside cellulose. In the next step, covalent bonds were formed from hydrogen bonds [2].

## 4. Conclusions

1. Lignin undergoes a silanization reaction with alkoxysilanes much easier than cellulose. Cellulose silanization efficiency without a catalyst is unlikely. Higher lignin reactivity when compared with cellulose is caused by the presence of more acidic hydroxy groups. Moreover, the amorphous character of lignin makes its reactivity easier in contrast to cellulose, which presents a crystalline and amorphous structure. The reactivity was proven by FT-IR and NMR analyses. Bonds between lignin and MTMOS were identified in the reaction without catalysts. No significant influence of catalyst activity on lignin silanization reaction was observed.
2. Depending on various catalysis mechanisms, diverse efficiency of cellulose silanization was observed. The highest efficiency of silanization was observed for the reaction where Al(acac)_3_ was used. These results were confirmed by FT-IR and AAS analyses. The silanization mechanism with the transition state of H_2_O and Al(acac)_3_ appeared to be more efficient than commonly applied acid catalysis.
3. The durability of silanization of cellulose and lignin in the presence of Al(acac)_3_ was confirmed. T^1^, T^2^ or T^3^ structures on NMR spectra were observed after leaching. The results of AAS analysis confirmed, additionally, the effective silanization, where the content of silicone was higher after leaching.

## Figures and Tables

**Table 1:** Content of reaction mixtures.

Sample	Material	Methyltrimethoxysilane (MTMOS)	Ethanol	Catalyst
C	cellulose	−	−	-
C–M	cellulose	+	+	-
C–M–Al	cellulose	+	+	aluminum acetylacetonate (Al(acac)_3_)
C–M–Ac	cellulose	+	+	acetic acid (AcOH)
L	lignin	−	−	-
L–M	lignin	+	+	-
L–M–Al	lignin	+	+	Al(acac)_3_
L–M–Ac	lignin	+	+	AcOH

**Table 2:** Comparison of characteristic band intensity to basic bands observed on FT-IR spectra for cellulose silanized with MTMOS.

Sample	Band Intensity Ratio				
	770/897 Si–CH_3_/C–H	1032/897 (Si–O/C–H)	1127/897 (Si–O–C/C–H)	1270/897 Si–CH_3_/C–H	3360/897 OH/C–H
C	—	—	—	—	3.72
C–M	0.38	7.45	6.17	1.34	6.53
C–M–Al	1.67	4.47	3.92	2.14	3.20
C–M–Ac	0.49	4.83	4.13	1.29	4.03

**Table 3:** Comparison of the lateral order index (LOI), total crystalline index (TCI) and hydrogen bond intensity (HBI) of cellulose silanized with MTMOS.

Sample	LOI	TCI	HBI
C	1.98	1.43	1.83
C–M	1.89	1.26	3.24
C–M–Ac	1.22	1.26	3.15
C–M–Al	1.33	1.26	2.54

**Table 4:** Comparison of characteristic band intensity to basic bands observed of FT-IR spectra for lignin silanized with MTMOS.

Sample	Band Intensity Ratio					
	777/1510 (Si–CH_3_/C–H_Ar_)	920/1510 (Si–OH/C–H_Ar_)	1032/1510 (Si–O/C–H_Ar_)	1127/1510 (Si–O–C/C–H_Ar_)	1270/1510 (Si–CH_3_/C–H_Ar_)	3450/1510 (OH/C–H_Ar_)
L	—	—	—	—	—	0.69
L–M	0.52	0.17	1.40	1.29	1.40	0.88
L–M–Al	0.77	0.14	1.89	1.44	1.51	0.94
L–M–Ac	0.89	0.18	1.98	1.82	1.73	0.91

**Table 5:** Atomic absorption spectroscopy (AAS) results.

Sample	Si (mg/g)	Sample	Si (mg/g)
C	0.00	L	0.00
C–M	3.60	L–M	61.22
C–M–Al	61.49	L–M–Al	71.72
C–M–Ac	34.94	L–M–Ac	84.07
C–W	0.00	L–W	0.00
C–M–W	0.54	L–M–W	57.81
C–M–Al–W	70.02	L–M–Al–W	108.2
C–M–Ac–W	16.05	L–M–Ac–W	76.43
